# Editorial: Postoperative management of Crohn's disease: One size does not fit all

**DOI:** 10.1002/ueg2.12381

**Published:** 2023-03-15

**Authors:** Eugeni Domènech, Míriam Mañosa, Margalida Calafat

**Affiliations:** ^1^ IBD Unit Gastroenterology Department Hospital Universitari Germans Trias i Pujol Catalonia and Centro de Investigación Biomédica en Red de Enfermedades Hepáticas y Digestivas (CIBEREHD) Badalona Spain; ^2^ Facultat de Medicina Universitat Autònoma de Barcelona Bellaterra Spain

**Keywords:** Crohn's disease, endoscopy, gastroenterology, inflammatory bowel disease, surgery

It was in the late eighties and early nineties that the Leuven's group led by Paul Rutgeerts published a series of landmark studies describing the natural history of postoperative Crohn's disease (CD). These authors introduced the concept of postoperative recurrence (POR) as defined by the development of disease‐related mucosal lesions at the neoterminal ileum after a “curative” ileocecal resection. They showed that this phenomenon occurs early after surgery in up to 80% of patients within the first 12 months and that there is a clear correlation between the severity of these lesions seen at ileocolonoscopy and the risk of developing symptoms (clinical POR), which in some patients may lead to a new intestinal resection (surgical POR).^1^, ^2^, ^3^

Since then, many randomized, controlled studies have been performed to assess the efficacy of a number of drugs to prevent POR. To date, only thiopurines and anti‐TNF agents have been demonstrated to be useful in the prevention of early endoscopic POR.^4^

But bearing in mind that not all the patients will develop POR (and in a significant proportion of those who will, only intermediate lesions carrying a low risk of clinical and surgical POR will occur) and that the use of immunosuppressive therapies carries a risk of adverse effects, the best strategy after intestinal resection is still under debate.

Systematic prevention with thiopurines and anti‐TNF agents is supported by their demonstrated efficacy. Moreover, most of these patients already developed disease‐related complications that drove them to surgery; therefore, leaving them without any maintenance therapy is against the current therapeutic goals that promote intensive treatment in those patients at risk of disabling disease.

On the other hand, some authors propose the so‐called “endoscopy‐driven strategy”. Based on the benefits of treatment escalation in case of advanced endoscopic lesions,^5^ this strategy proposes early endoscopic monitoring and treatment with thiopurines or anti‐TNF agents only in case of endoscopic POR. Although a statistically underpowered study found no differences in the rate of endoscopic POR after 2 years between this strategy and systematic prevention,^6^ it is also true that there is a risk of being late to reverse mucosal damage, leading to persistence or progression of mucosal lesions in up to 30%–50% of patients.^7^

Finally, given that there are some epidemiological and clinical features that have been associated with a higher risk of early POR, the third strategy defends that the decision to start prevention or wait for endoscopic monitoring should be based on the presence or absence of risk factors. However, with the available risk factors in daily clinical practice, this strategy seems to be useless in improving postoperative outcomes.^8^ Recently, a prospective French research project on different POR issues found that the more risk factors, the higher the risk of endoscopic POR.^9^ As a consequence, these investigators decided to establish a decision‐making protocol of POR prevention based on the number of risk factors. Unfortunately, this “stratification strategy” led to similar rates of endoscopic POR between those patients with no, one, or more than one risk factor in whom no prevention, thiopurines, and anti‐TNF agents were started after surgery, respectively.^10^

In this issue of *United European Gastroenterology Journal*, Dragoni et al^11^ explore a somewhat different aspect of this complex clinical scenario. The Italian Group in Inflammatory Bowel Disease designed a retrospective study to assess whether primary prevention or endoscopy‐driven strategies work better in patients meeting only one risk factor for POR. Almost 200 adult CD patients who underwent ileocolic resection, met only one out of five well‐established POR risk factors (i.e. previous intestinal resection, >50 cm small bowel resection, fistulising phenotype, history of perianal disease, or active smoking), and had at least one available ileocolonoscopy 6–12 months after surgery were included. The main endpoint was endoscopic POR within 12 months after surgery. The authors did not observe any difference in the rates of endoscopic POR (defined by a Rutgeerts score > i2a), severe endoscopic POR (Rutgeerts score i4), and clinical POR between the two study groups.

Although the study has some gaps related to its retrospective design (lack of central review of endoscopic examinations, different timings for endoscopic assessment, and different drug therapies in the primary prevention group), the observed results warrant a prospective evaluation of new decision‐making approaches in the postoperative setting of CD. Both primary prevention and endoscopy‐driven therapy strategies seem to be useful in the suitable patient…but how do we identify the patient? Maybe it is time to bring in histologic^12^, ^13^ and microbiological^14^ data for decision‐making.

## CONFLICTS OF INTEREST STATEMENT

The corresponding author confirms on behalf of all authors that there have been no involvements that might raise the question of bias in the work reported or in the conclusions, implications, or opinions stated. Eugeni Domènech has served as a speaker or has received research or educational funding or advisory fees from AbbVie, Adacyte Therapeutics, Biogen, Celltrion, Galapagos, Gilead, Janssen, Kern Pharma, MSD, Pfizer, Roche, Samsung, Takeda, Tillots; Míriam Mañosa has served as a speaker and has received research or educational funding from MSD, AbbVie, Takeda, Janssen, Faes Farma, Ferring and Pfizer; Margalida Calafat has served as a speaker for Takeda, Janssen, Faes Farma, and MSD.

## Data Availability

Author elects to not share data.
